# Evaluating the Efficacy of a 5‐Min Music Listening Intervention for State Anxiety Reduction in College Students: A Randomized Controlled Trial

**DOI:** 10.1002/hsr2.70590

**Published:** 2025-03-18

**Authors:** Jameel Soqia, Laila Yakoub‐Agha, Mohammad Basheer Alameer, Lujain Nahas, Lama Mohamad, Ibrahim Antoun, Caroline Almohsen, Samer Mohsen

**Affiliations:** ^1^ Faculty of Medicine Damascus University Damascus Syria; ^2^ Department of Cardiovascular Sciences University of Leicester Leicester UK; ^3^ Department of Clinical Psychology, Faculty of Health Sciences Damascus University Damascus Syria; ^4^ Department of Audiology, Faculty of Health Sciences Damascus University Damascus Syria

**Keywords:** mental health, music, spielberger state anxiety inventory (STAI‐S), state anxiety

## Abstract

**Objective:**

The primary objective of this research was to investigate the impact of a brief 5‐min period of listening to a standardized piece of music (Oriental Jazz) on state anxiety among Syrian college students, providing insights into the potential effectiveness of music as a pre‐lecture anxiety reduction tool.

**Methods:**

A two‐phase randomized controlled trial conducted in Damascus, Syria. The participants were college students aged 18–24 who met specific criteria and were randomly assigned to either a music intervention group or a control group. State anxiety was measured pre‐ and post‐intervention using the spielberger state anxiety inventory (STAI‐S).

**Results:**

In total, 69 participants were included in this study. The mean score decreased non‐significantly in the intervention group (37.9–36.8, *p* = 0.258) and the control group (46–43.6, *p* = 0.444). The changes in the anxiety score on phase 2 were insignificant between both study arms (*p* = 0.622). These results indicate that music has no significant effect on STAI scores.

**Conclusion:**

Our study did not find evidence supporting the anxiety‐reducing effects of a brief exposure to a unified piece of music (Oriental Jazz). Given these findings, this intervention does not appear to have benefits for reducing anxiety when applied before lectures. Future investigations should consider personalized music interventions, duration, and individual preferences.

## 1 Introduction

Anxiety is a feeling of apprehension, tension, or uneasiness caused by anticipation of danger, which may be internal or external [1]. It can also be described as an adverse effect regarding unpredictable and unavoidable future danger accompanied by heightened vigilance and physiological symptoms of tension [2]. Anxiety serves as a defense mechanism, enabling the organism to avoid or reduce harm [3], but experiencing inappropriate or excessive anxiety can lower the quality of life [4]. Anxiety disorders comprise a group of heterogeneous disorders with different causes and outcomes [5]. A type of anxiety is state anxiety, which is transient in the appearance of the symptoms above [6]. Most of the population can experience state anxiety, but there are many differences among individuals regarding frequency, duration and severity [5]. Anxiety disorders are typically managed through a combination of psychotherapy, particularly cognitive behavioral therapy (CBT), and lifestyle changes such as regular exercise, relaxation techniques like yoga, and a balanced diet can support treatment [7].

Music listening (ML), an accessible and cost‐effective alternative treatment for anxiety, can be easily integrated into daily life [8]. Unlike music therapy, ML does not require a qualified therapist and can be self‐administered. It involves presenting prerecorded or live music to individuals or groups [9]. ML interventions have shown high adherence rates, enjoyment, and low drop‐out rates, making them appealing to anxiety management [10]. ML can be delivered remotely, which makes it easy and valuable [9]. ML has demonstrated associations with reduced physiological indicators of anxiety, including heart rate and blood pressure [11]. ML modulates the stress response by lowering cortisol levels [12]. Extensive literature highlights ML's potential for affect regulation, encompassing mood, emotion, and arousal [13, 14]. Groarke and Hogan [15, 16] propose an adaptive model linking ML to well‐being, incorporating social cognitive theory [17]. This model emphasizes on both affective experiences and the significance of social and eudemonic factors in promoting well‐being.

Anxiety is a prevalent issue among college students, often exacerbated by the demands of academic life. High levels of anxiety can negatively affect cognitive function, learning, and overall well‐being, making it crucial to identify effective strategies to alleviate this condition in educational settings [18]. Numerous studies have reported that listening to music can have therapeutic effects, including the reduction of anxiety and stress levels [8]. In a systematic review and meta‐analysis, researchers investigated the impact of ML interventions on naturally occurring state anxiety. The results revealed that ML had a significant enormous effect in alleviating anxiety across various groups [9]. However, they indicated that further research is needed [9]. However, the outcomes have been inconsistent, with some studies demonstrating significant benefits while others suggest minimal to no effect, particularly when applied in diverse and uncontrolled environments like classrooms or lecture halls [19, 20].

The primary objective of this research was to investigate the impact of a brief 5‐min period of listening to a standardized piece of music (Oriental Jazz) on state anxiety among Syrian college students. A unified musical piece was selected to ensure consistency and feasibility in its application as a potential pre‐lecture intervention across the university. To achieve this, we designed a randomized controlled trial with two groups: a music intervention group (Group A) and a control group (Group B).

## 2 Methods

### 2.1 Study Design

The objective of this method is to examine whether a 5‐min session of listening to Oriental Jazz before lectures can significantly lower state anxiety among students. To evaluate this, a randomized controlled trial was conducted with participants assigned to either a music intervention group (Group A), which listened to Oriental Jazz, or a control group (Group B), which did not receive the musical intervention. This design enables a systematic comparison of anxiety levels between the two groups, providing insights into the potential effectiveness of music as a pre‐lecture and pre‐exam anxiety reduction tool. The decision to use a standardized piece of music, specifically Oriental Jazz, was based on its potential to create a calming and culturally relevant auditory environment with the same culture of our participants (rather than using a different cultural music such as westeren classic music or normal jazz), as this may reduce state anxiety among Syrian college students. Oriental Jazz was chosen because it combines familiar cultural elements with jazz improvisation, creating a unique musical experience that is both engaging and soothing. Additionally, Oriental Jazz was used because it is not studied before, nor any oriental music. This style was considered suitable for a diverse student population as it avoids lyrics that may distract or evoke strong emotions and offers a balance of rhythm and melody that can promote relaxation.

Given the large number of students and the logistical challenges of catering to individual musical preferences, a unified musical piece ensures a consistent intervention that can be feasibly applied in a university setting. This design was chosen to address the practical constraints faced by low‐income countries, where resources for individualized interventions are limited. Our literature review revealed no prior studies with a similar design, highlighting the innovative nature of our research. Additionally, we selected oriental jazz for its cultural relevance to our participants, ensuring the music is familiar and potentially more effective. By exploring the effects of oriental jazz, we aim to contribute new insights to the literature, as this genre has not been previously studied in this context.

We selected a 5‐min duration for the music intervention to ensure it could be feasibly integrated into pre‐lecture or pre‐exam schedules without causing significant disruption, while also addressing the gap in literature regarding the effects of short music sessions, particularly with culturally relevant music like oriental jazz.

### 2.2 Participants

The sample represents college students aged 18–24 years at the faculty of health sciences at Damascus University who have not consumed any alcoholic beverages in the last 24 h, have not taken any medications that affect cognitive performances (such as antidepressants), have not previously suffered from a head injury which caused a concussion, and do not suffer from major depressive disorder or extreme sleep disturbances. All faculty students were invited to join the study voluntarily by filling out the online survey, which consists of explicit consent of participation and a request for (name, age, contact info, alcohol consumption in 24 h, previous head injuries, specific medication intake). The invitation survey was administered on Google Docs and sent to the official faculty group by the dean. Before the study, students were intentionally unaware of the main intervention of study's purpose or any specific details, as they were told only the study is about the music effect on anxiety levels. This approach was taken to ensure blinding and minimize any potential personal bias. We explained the entire research to the participants only after the study concluded and we have taken their written consent before the study and after the study to continue analyzing the data.

The sample size was calculated using the G‐power version (3.1.9.4). Based on previous research, we estimated that the effect size is 0.3%, the power is 80%, and the alpha error is 0.05. Thus, the total sample required was 69. One hundred and two responses to our invitation were initially recorded. However, 28 responses were excluded because of missing data or not meeting the sample criteria. Then, five more participants were excluded as they had high perceived stress and poor sleep quality in the last few days. Thus, the final number of students who had completed the study was n = 69.

### 2.3 Randomization

In this study, participants were assigned randomly to either Group A or Group B in a 34:35 ratio. All participants completed both phases of the trial. Before the study commenced, the primary researcher utilized an online tool called the Research Randomizer to randomly allocate the participants to one of the two groups in a random order. To achieve this, 69 unique codes were generated and randomly assigned to either Group A or Group B. Then, a blinded researcher placed the code into a container without retaining any specific order. Afterwards, the leading researcher randomly selected one code for each participant, ensuring that all 69 participants were equally distributed between the groups and had an equal chance to be in either group: Group A (music group) and Group B (control group).

### 2.4 Procedures

The trial was on two phases in the same day:

- Phase 1 (Basline): All participants completed the perceived stress scale PSS and the Pittsburg Sleep Quality Index PSQI questionnaires before starting the tests, which took approximately 15 min. Then participants were asked to complete the spielberger state anxiety inventory (STAI‐S). The whole phase took almost 20–25 min to be finished then we asked students to start Phase 2.
- Phase 2: Participants of group A were asked to move to other rooms and listen to a 5‐min Oriental Jazz music piece (Will soon be a woman—Ibrahim Maalouf, 5 min and 32 s) administered to them via wired earphones without visual distractions in addition to volume setting to 75% level on a scale from 0 to 100 for all participants. Subsequently, they were asked to complete the STAI‐S. While participants of group B were asked to go to different rooms and complete the STAI‐S after 5 min of silence and no activity.

**Figure 1:**
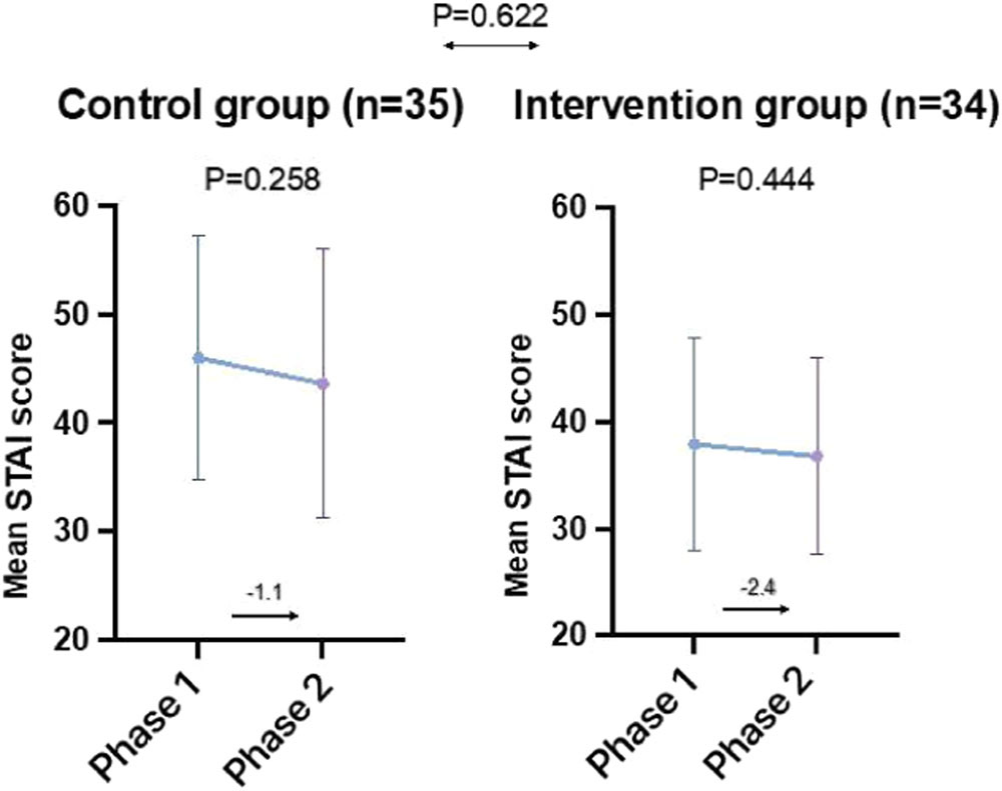
Comparative analysis of state anxiety reduction between intervention and control groups. STAI: State‐trait anxiety inventory.

Figure 1 clarifies the procedures of the whole study.

## 3 Measurements

- *The perceived stress scale (PSS):* This self‐administered questionnaire contains ten items measuring the relative stress level over 1‐month. The score of this self‐reporting subjective tool ranges from 0 to 40. A previously validated Arabic version of the scale was used in this study [21].
- *The Pittsburg Sleep Quality Index (PSQI)* is a 19‐item self‐administered questionnaire to assess sleep quality and disturbances over 1 month. It comprises seven items: (1) Sleep duration, (2) sleep latency, (3) sleep disturbance, (4) daytime dysfunction due to sleepiness, (5) sleep efficiency, (6) overall sleep quality, and (7) sleep medication use. Each item scores 0–3, and the sum of each item results in one global score. The higher the global score, the worse the overall quality of sleep. A previously validated Arabic version of the scale was used in this study [22].
- *Spielberger State Anxiety Inventory (STAI‐S):* This scale comprises 20 statements, with subjects indicating their feelings at a specific moment. This scale assesses anxiety intensity resulting from stressful situations. Participants rate each question on a 4‐point scale: not at all, somewhat, moderately, or very much so—the possible scores for the STAI‐S range from a minimum of 20 to a maximum of 80. A previously validated Arabic version of the scale was used in this study. The present study obtained a Cronbach's *α* of 0.88 for the STAI‐S, suggesting an internal solid reliability [23].

### 3.1 Ethical Considerations

The terms and conditions of the two‐phase trial were explained in the participation survey generally, and informed consent was obtained about the intervention from the participants at this point. After the conduction of the study, we explained the entire research to the participants, and we have taken their written consent again. The study was approved by the ethical committee at Damascus University Faculty of Health Sciences (ID number: FHS‐140724‐274, date: 24/10/2024) and was conducted according to the moral standards laid down in the Helsinki II Declaration regarding informed consent, voluntariness, and anonymity. The questionnaires used no names or personal information that would compromise the personal safety of the participants. They were informed of the freedom to continue or quit the experiment.

### 3.2 Statistical Analysis

All analyses were performed using IBM SPSS Statistics for Windows, version 25 (IBM Corp., Armonk, NY, USA). Continuous variables are expressed as mean and standard deviation. Categorical variables are expressed as counts and percentages (%). Results included demographic data, pre‐tests, and post‐test results for STAI‐S, PSS, and PSQI scores. Paired and unpaired student tests were utilized to assess the changes in STAI scores between baseline and day two between study arms after ensuring the data followed a normal distribution using the Shapiro–Wilk test. A *p*‐value of < 0.05 is considered statically significant. Cohen's *d* was used to measure All effect sizes.

## 4 Results

### 4.1 Demographic Characteristics

In total, 69 participants were included in this study. As outlined in Table 1, 59 participants were female (84.3%), none of the participants had any head injuries or experienced unconsciousness in the last month, and none of the participants have taken any medication that affects their memory or mental health. Moreover, 85.5% of participants had moderate stress, and 76.5% had a moderate sleep quality. Further details, characteristics and participant differences across the three regions are outlined in Table 1.

**Table 1:** The study sample characteristics.

Variables		Intervention group (*n* = 34)	Control group (*n* = 35)	Total (*n* = 69)
Gender	Males	3 (8.8%)	7 (20%)	10 (14.5%)
	Females	31 (91.2%)	28 (80%)	59 (85.5%)
Head injuries or experienced unconsciousness	No	34 (100%)	35 (100%)	69 (100%)
Alcohol drinking in the last 24 h	No	34 (100%)	35 (100%)	69 (100%)
Medication in the last 24 h	No	34 (100%)	35 (100%)	69 (100%)
Stress levels (PSS)	Low perceived stress	8 (23.5%)	2 (5.7%)	10 (14.5%)
	Moderate perceived stress	26 (76.4%)	33 (94.3%)	45 (85.5%)
	High perceived stress	3 (excluded)	2 (excluded)	5 (excluded)
Sleep Quality (PSQI)	Good	11 (32.4%)	5 (14.7%)	16 (23.5%)
	Moderate	23 (67.6%)	29 (85.3%)	52 (76.5%)
	Poor	3 (excluded)	2 (excluded)	5 (excluded)

## 5 Music Effect on State Anxiety

Figure 1 compares state anxiety mean scores between the intervention and control groups. The mean score decreased non‐significantly in the intervention group (*M* = 37.9–36.8, SD = 9.9–9.2, *p* = 0.258) and the control group (*M* = 46–43.6, SD = 11.2–12.3, *p* = 0.444). The changes in the anxiety score on phase 2 were insignificant between both study arms (*p* = 0.622). These results indicate that music has no significant effect on STAI scores.

## 6 Discussion

The current study investigated the impact of 5 min of listening to a standardized piece of music (Oriental Jazz) on state anxiety among college students. However, the results did not show a significant decrease in state anxiety scores in the intervention or control groups. The demographic characteristics showed a similar distribution in both groups, even though most participants were female.

The current study's findings contrast with other research that has found music to have a positive impact on anxiety levels. For instance, a systematic review and meta‐analysis found that music therapy had a medium‐to‐large effect on stress‐related outcomes [8]. However, that systematic review focused on music therapy, which involves personally tailored music interventions initiated by a trained and qualified music therapist, distinguishing it from other music interventions such as ML. Other research on anxiety has revealed that listening to prerecorded music could positively impact preoperative anxiety [24]. However, previous studies often involved music pre‐selected by participants, allowing for personal preferences that may have influenced the outcomes. In contrast, the current study used a standardized piece of Oriental Jazz music, chosen to ensure consistency across a large group of students. This may have impacted the results differently, highlighting the complexity of using a unified musical intervention for anxiety reduction.

Compared to other studies, our findings contradict those of Huang et al., who found that neutral music alleviated state anxiety [25]. However, their study did not focus on the duration of the music intervention, which could explain the discrepancy in the results. Additionally, a survey by Tolley and Vick indicated that even short durations of ML may reduce anxiety in undergraduate students [26]. However, their study used music pre‐selected by the participants, which could have influenced the results. Moreover, a study found that participants who listened to soothing music showed significantly lower tension and state anxiety levels than those who listened to stimulative music when the music was unpreferred [27]. This suggests that personal preference and familiarity with the music might play a role in its effectiveness in reducing anxiety. Therefore, while music can impact state anxiety, the type of music and the context in which it is listened to can significantly influence its effectiveness.

**Implications:** Despite the initial hypothesis, the results did not reveal a significant decrease in state anxiety scores for either the intervention or control groups. Several implications can be drawn from these findings:

- **Limitations for use before lectures:** Given the lack of significant anxiety reduction, the use of a standardized music session before lectures may not be an effective strategy for lowering anxiety across a diverse student population. Individual preferences and responses to music vary widely, making a unified approach less practical for large groups.
- **Complexity of music's impact on anxiety:** The relationship between music and anxiety is multifaceted. While some studies have reported positive effects, our study did not replicate these findings. Personal preferences, individual differences, and contextual factors likely contribute to the variability in music's impact on anxiety.
- **Pre‐selected versus unified music:** Previous research has often used pre‐selected music, allowing the participants to choose familiar tunes. In contrast, we exposed participants to a unified piece of music. This might have influenced participants' responses, affecting anxiety levels differently.

**Limitations:** This study has several limitations. First, the study only measured the immediate effects of the music intervention on state anxiety. Therefore, it does not provide insight into the long‐term effects of such interventions. Second, this study was conducted among college students. Thus, the results could not be generalizable to other populations and age groups. Additionally, sleep disturbances and anxiety disorders existed in this study sample with a moderate percentage, as in the context of the Syrian population, it is expected to have a moderate rate, as previous research shown during a crisis [28], so this limits the ability to generalize the result of this study in healthier populations. Another limitation of our study is the inability to measure individual sound sensitivity due to the low‐resource environment and lack of appropriate equipment, which may have affected the uniformity of the music intervention's impact.

Lastly, the study did not consider other factors that could influence state anxiety, such as memory ability, which is associated with anxiety. It would also be beneficial to conduct follow‐up studies to examine the long‐term effects of music interventions on state anxiety.

## 7 Conclusion

Our study did not find evidence supporting the anxiety‐reducing effects of a brief exposure to a unified piece of music (Oriental Jazz). However, this contrasts with other research that highlights music's potential to alleviate anxiety. Given these findings, this intervention does not appear to have benefits for reducing anxiety when applied before lectures. Future investigations should consider personalized music interventions, duration, and individual preferences. Ultimately, the type of music and the context in which it is experienced significantly shape its effectiveness in reducing anxiety.

## Author Contributions

**Jameel Soqia:** conceptualization, data curation, visualization, formal analysis, writing – original draft, methodology, investigation, project administration, writing – review and editing, validation, software, resources. **Laila Yakoub‐Agha:** conceptualization, data curation, visualization, writing – original draft, methodology, investigation, software, validation, resources, project administration. **Mohammad Basheer Alameer:** data curation, investigation, writing – original draft, writing – review and editing, validation, software, resources. **Lujain Nahas:** data curation, investigation, validation, resources, software. **Lama Mohamad:** data curation, investigation, software, validation, resources. **Ibrahim Antoun:** data curation, formal analysis, writing – original draft, writing – review and editing, software, resources. **Caroline Almohsen:** conceptualization, visualization, supervision, writing – review and editing, resources, investigation, methodology, project administration. **Samer Mohsen:** methodology, investigation, supervision, project administration, writing – review and editing, conceptualization, visualization, validation, resources.

## Conflicts of Interest

The authors declare no conflicts of interest.

## Transparency Statement

The lead author Jameel Soqia affirms that this manuscript is an honest, accurate, and transparent account of the study being reported; that no important aspects of the study have been omitted; and that any discrepancies from the study as planned (and, if relevant, registered) have been explained.

## Data Availability

The data that support the findings of this study are available from the corresponding author upon reasonable request.
